# Behaviour of Hybrid Fibre-Reinforced Ternary Blend Geopolymer Concrete Beam-Column Joints under Reverse Cyclic Loading

**DOI:** 10.3390/polym14112239

**Published:** 2022-05-31

**Authors:** Veerappan Sathish Kumar, Namasivayam Ganesan, Pookattu Vattarambath Indira, Gunasekaran Murali, Nikolai Ivanovich Vatin

**Affiliations:** 1 Faculty of Civil Engineering, Architecture and Geodesy, University of Split, 21000 Split, Croatia; 2 Department of Civil Engineering, National Institute of Technology Calicut, Kerala 673601, India; ganesan@nitc.ac.in (N.G.); indira@nitc.ac.in (P.V.I.); 3 Peter the Great St. Petersburg Polytechnic University, 195251 St. Petersburg, Russia; murali_22984@yahoo.com (G.M.); vatin@mail.ru (N.I.V.)

**Keywords:** beam-column joint, ductility, energy absorption, geopolymer concrete, hybrid fibre, reverse cyclic loading, shear strength, ternary blend

## Abstract

Beam–column joints are extremely vulnerable to lateral and vertical loads in reinforced concrete (RC) structures. This insufficiency in joint performance can lead to the failure of the whole structure in the event of unforeseen seismic and wind loads. This experimental work was conducted to study the behaviour of ternary blend geopolymer concrete (TGPC) beam-column joints with the addition of hybrid fibres, viz., steel and polypropylene fibres, under reverse cyclic loads. Nine RC beam-column joints were prepared and tested under reverse cyclic loading to recreate the conditions during an earthquake. M55 grade TGPC was designed and used in this present study. The primary parameters studied in this experimental investigation were the volume fractions of steel fibres (0.5% and 1.0%) and polypropylene fibres, viz., 0.1 to 0.25%, with an increment of 0.05%. In this study, the properties of hybrid fibre-reinforced ternary blend geopolymer concrete (HTGPC) beam-column joints, such as their ductility, energy absorption capacity, initial crack load and peak load carrying capacity, were investigated. The test results imply that the hybridisation of fibres effectively enhances the joint performance of TGPC. Also, an effort was made to compare the shear strength of HTGPC beam-column connections with existing equations from the literature. As the available models did not match the actual test results, a method was performed to obtain the shear strength of HTGPC beam-column connections. The developed equation was found to compare convincingly with the experimental test results.

## 1. Introduction

Reinforced concrete (RC) constructions are becoming more popular as a result of recent earthquakes in different parts of the world. In RC multi-storey frames subjected to unstable wind and seismic loads, beam-column joints are the most critical sites in the structure. During earthquakes, shear failure is the most typical kind of brittle failure and is undesirable. These joints need to be constructed to handle shear pressures accompanied by substantial deformations in order to avoid the rapid collapse of the entire structure. The joint shear strength of the structure can be improved by using transverse reinforcement. As a result of transverse shear reinforcement in the connections, placing and compacting the concrete becomes difficult [1]. Hence, finding new ways to increase joint shear strength is essential in light of current circumstances.

To minimise transverse reinforcement congestion at beam-column connections, FRC (fibre-reinforced concrete) is an excellent and practical solution [2,3,4,5]. The structural integrity of FRC is mainly based on the properties of the fibres, such as fibre geometry, the orientation of the fibre in the concrete, the type of fibre and the volume fraction of the fibre [6,7,8,9,10]. Several studies have addressed that incorporating fibres in concrete can improve properties such as impact resistance, flexural strength, thermal resistance, fatigue resistance and ductility [11,12,13,14,15]. Brandt [16] evaluated the orientation effect of parallel fibre systems and concluded that the maximum fracture energy was due to the fibres being subjected to axial tension. The effect of fibres with different aspect ratios was inspected by Mohammadi et al. [17]. They noted that concrete featuring short fibres performed better in terms of strength and workability than concrete featuring long fibres. The tensile behaviour of FRC with various aspect ratios and fibre volumes was examined by Yazici et al. [18]. They reported that the concrete’s flexural strength was increased by up to 54% after fibres were introduced.

In order to achieve a level of performance that is superior to that of FRC, hybrid fibre, a relatively new innovation, involves the mixing two or more kinds of fibres in concrete [19,20,21,22]. With the use of hybrid fibres, the characteristics of concrete can be improved in both fresh and hardened conditions [23,24]. Souvik et al. [25] studied the rheological and mechanical properties of steel and coir hybrid fibre-reinforced concrete (HFRC). They noted that, with a 2% hybrid fibre concentration, the split tensile and flexural strength of the concrete rose by 18.36% and 24.87%, respectively, but the compressive strength was only marginally improved. According to the findings of Abubaker and Yanmin [26], the integration of glass and polypropylene fibres into HFRC increases the concrete’s impermeability, resulting in a longer life expectancy for the structure. Due to the improved post-cracking properties of metallic fibres, hybrid fibres with both metallic and non-metallic components are the most popular. In contrast, non-metallic micro fibres arrest microcracks in the concrete [27,28,29].

Numerous researchers have examined the consequences of fibre hybridisation on the structural behaviour of different concretes such as conventional cement concrete (CC), high-performance concrete (HPC), lightweight concrete (LWC), high strength concrete (HSC), self-compacting concrete (SCC) and geopolymer concrete (GPC) [30,31,32,33,34]. Ganesan et al. [1] performed an experimental investigation to study the effect of fibre hybridisation on HPC beam-column joints subjected to reverse cyclic loading conditions. They found that using hybrid fibres significantly increased the ductility and energy dissipation capacity of the concrete. The structural performance of beam-column connections using hybrid fibres was observed by Iqbal et al. [35], who noted that combining synthetic fibres improved behaviours such as the steel reinforcement strain, ductility and stiffness degradation rate. Many researchers have also reported investigations on beam-column joints with mono-fibres to study the structural behaviour of reinforced concretes such as CC, SCC, GPC, etc., [36,37,38]. All of these studies on beam-column joints with mono and hybrid fibres were limited to various types of concretes such as CC, HPC, SCC and GPC. However, the effect of fibre hybridisation on blended GPC beam-column joints has not yet been reported.

Inorganic geopolymer is one of the more promising materials in the context of efforts to replace conventional cement in concrete in order to minimise the carbon footprint associated with its production [39]. Studies showed that the use of GPC in the construction industry could reduce greenhouse gas emissions by 25–70% [39]. GPC successfully utilises industrial by-products, including fly ash, GGBS, kaolin, silica fumes, etc., as silica and alumina rich source materials. The silica and alumina in the source materials are activated by an alkali activator to form the binder in concrete [40]. Concrete manufactured using this alkali-activated binder possesses improved characteristics such as durability, strength, fire resistance and freeze-thaw resistance [40,41,42]. In a recent development in the field of GPC, geopolymer composites were developed using artificial intelligence, and their potential is increasingly attracting the attention of researchers [43,44].

Raj and Ramachandran [45] studied the effect of hybrid fibre on fly ash-based geopolymer concrete and observed that using hybrid fibres significantly improved the ductility of beams up to a maximum of 43%. The incorporation of hybrid fibres in TGPC beams was investigated [46,47]. It was noted that the addition of fibres in a hybrid form improves the ductility behaviour of geopolymer concrete beams and changes the mode of failure from shear to flexure. The incorporation of hybrid fibres on the ductility and impact resistance of GGBS-based GPC was studied and it was concluded that the ductility index was enhanced efficiently [48,49,50]. The impact resistance of GPC was also improved significantly with the introduction of fibres in mono and hybrid forms, changing the mode of failure from brittle to ductile [51].

GPC produced with blended source materials exhibits increased and high early strength. Vijayasarathy et al. [52] presented the properties of binary blend geopolymer concrete and revealed that the addition of GGBS with fly ash to GPC increased the strength performance up to 25%. The effect of adding sugarcane bagasse ash as a partial alternative for GGBS in GPC was investigated by Kathirvel et al. [53], who found that the replacement was effective in terms of improving the fresh and hardened properties up to a maximum of 40%. The durability of ternary blend geopolymer concrete (TGPC) with hybrid fibres was investigated by Sathish et al. [54], who found that the experimental results were within limits. All of these works were limited to the mechanical and durability properties of blended GPC. Hence, this work was carried out to investigate the structural parameters of hybrid fibre-reinforced ternary blend geopolymer concrete (HTGPC) beam-column joints. Furthermore, an effort was made to predict the shear strength of HTGPC beam-column joints in order to gain a deeper understanding of their shear strength for the purpose of designing structural elements.

## 2. Experimental Programme

### 2.1. Materials

#### 2.1.1. Source Materials

The source materials used in this experiment included low calcium class F fly ash, GGBS and metakaolin (MK). Fly ash from Mettur Thermal Power Station in Tamil Nadu (India), which met IS 3812:2003 [55], was used as the primary binder in this formulation. It has a dark grey colour and is mostly composed of alumina and silica at the percentages of 27.7 and 55.36, respectively. Its average particle size is 75 microns, and it has a specific gravity of 2.30.

Fly ash was substituted with GGBS compliant with BS 6699:1992 [56] collected from a local supplier. It is an off-white-coloured powder that mainly consists of 37.04% calcium oxide, 32.49% silica and 20.86% alumina. This material has a specific gravity of 2.88 and a particle size distribution of 30 microns.

For the ternary blend binder material, metakaolin (MK) was employed as the third precursor to the source material. MK has an average particle size of 2–3 microns and a cream-coloured appearance. It mostly contains silica and alumina at the percentages of 56.64 and 42.38, respectively, and has a specific gravity of 2.56. The complete morphological and chemical composition data of the source materials were presented in the previous studies conducted by the authors [54,57]. The main motive for selecting these source materials was based on the morphological characteristics which would achieve a denser concrete for improved durability [54].

#### 2.1.2. Fine and Coarse Aggregates

Fine aggregate (M-Sand) with a maximum particle size of 4.75 mm according to Zone II of IS 383:1970 (reaffirmed 2002) [58] was utilised. Crushed stone with a maximum particle size of 12.5 mm was used in the mix as the coarse aggregate.

#### 2.1.3. Activator

Sodium silicate solution (Na_2_SiO_3_) containing 28 percent SiO_2_, 8 percent Na_2_O and 64 percent H_2_O was used to activate the alumino-silicate binder. Sodium hydroxide (NaOH) pellets of 99 percent purity were added along with Na_2_SiO_3_ to prepare the alkaline activator.

#### 2.1.4. Superplasticiser and Water

The workability was enhanced by using a water-reducing additive called Conplast SP 430. Normal potable water obtained from the laboratory was used for the mixing of TGPC.

#### 2.1.5. Fibres

**Figure 1:**
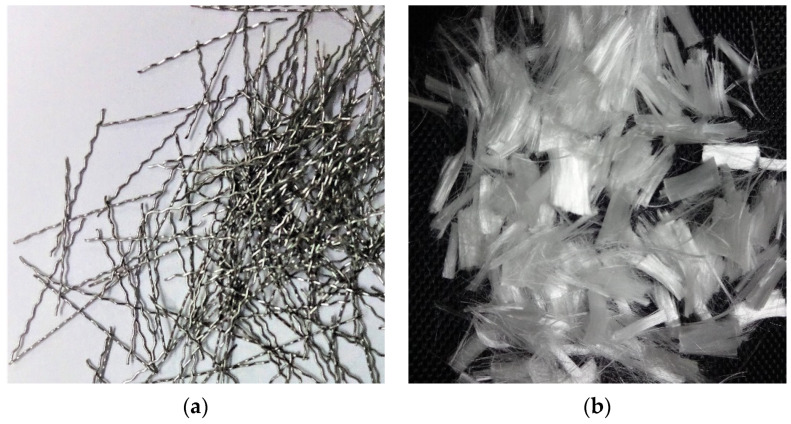
(**a**) Steel fibres and (**b**) polypropylene fibres.

In this investigation, the hybrid fibres used were (i) 30 mm long crimped steel fibres (SF) with a diameter of 0.45 mm and (ii) 12 mm long polypropylene fibres (PF) with a diameter of 40 microns. The polypropylene and steel fibres had a tensile strength of 600 MPa and 800 MPa, respectively. Figure 1 displays the steel and polypropylene fibre samples that were used in this study.

### 2.2. TGPC Mix Proportions

For the M55 grade TGPC, Rangan’s standards [59] were followed to determine the ingredients for the mixture proportions. Trial and error led to the current mix ratio, given that there is currently no standard way to create GPCs. The TGPC mixture design was established in a previous investigation performed by the authors [57,60]. As part of the TGPC, an alkaline activator to binder ratio of 0.3:1 was used. The binders were composed of 30 vol% fly ash, 25 vol% GGBS and 15 vol% MK. At 14 M sodium hydroxide, the ratio of added water to binder remained constant at 0.2 for all mix proportions studied. When conducting this experiment, the sodium silicate to sodium hydroxide ratio was maintained at 2.5. The mixture was given a boost in terms of workability with superplasticiser (SP) by adding 1.5 percent of the weight of the binder. Eight mixes with various volume percentages of steel and polypropylene fibres were considered in this work and compared with TGPC without fibres. The volume of steel fibre (SF) was varied by 0.5 vol% and 1.0 vol%, whereas the volume polypropylene fibre (PF) was varied by four volume percentages of 0.1 vol%, 0.15 vol%, 0.2 vol% and 0.25 vol%. Further details on the proportions of the fibres and their designated mix are presented in Table 1.

### 2.3. Specimen Details

**Figure 2:**
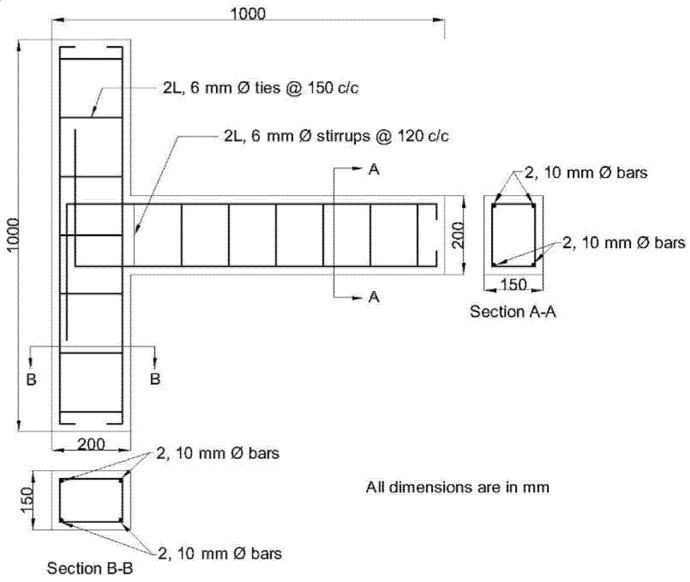
Details of the beam-column joint.

As part of this experiment, nine external beam-column joints with cross-sectional dimensions of 150 × 200 mm were cast and examined. The beam was 800 mm long, and the height of the column was 1000 m. The column and beam were reinforced with four 10-mm HYSD bars each. The column and beam stirrups, which were both 6 mm in diameter, were also made of HYSD bars. The reinforcing bars utilised in the project have the following mechanical characteristics, which are shown in Table 2. The joint was designed based on the principles of the strong column-weak beam concept, where the column-to-beam flexural strength ratio at the junction is estimated to be 2.2. The reinforcing details and measurements are provided as per the standards of the ACI-ASCE Committee 352 [61]. Figure 2 provides further detail on the overall size of the beam-column joint and its reinforcement.

### 2.4. Casting and Curing of Specimens

**Figure 3:**
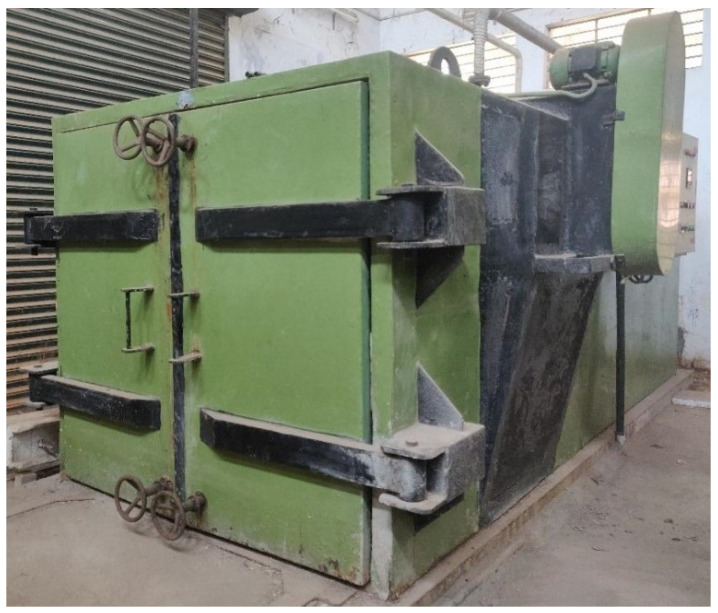
Steam curing chamber.

The beam-column joints were cast using a horizontal tilting drum concrete mixer, whereby the powder components were initially mixed with M-sand and coarse aggregates. To make the alkaine liquid, the sodium silicate solution was combined with 14 M sodium hydroxide solution 24 h before casting [24]. In the mixer, the alkaline liquid was added and mixed thoroughly until a homogeneous mixture was achieved. To ensure the even dispersion of the polypropylene fibres, the fibres were introduced before adding the alkaline liquid. To prevent the distortion of the steel fibres, these were added to the mixture at the very end. A needle vibrator was used to compact the mixture into a steel mould with reinforcing bars. After 24 h of casting, the specimens were transferred to a steam curing chamber (Figure 3) for curing at 60 °C with a plastic sheet on top [46]. After 24 h inside the curing chamber, the samples were taken out and placed at ambient temperature until testing.

### 2.5. Test Setup

**Figure 4:**
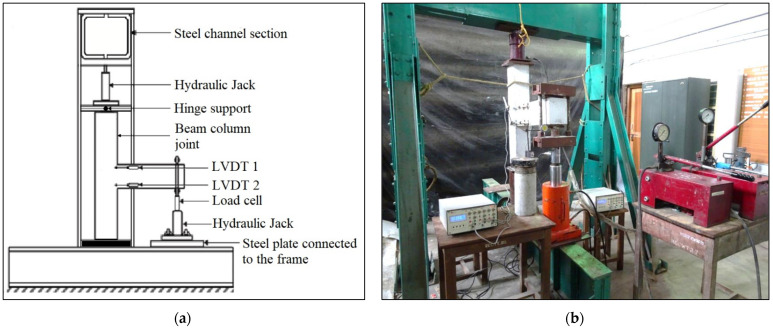
(**a**) Schematic diagram of the test setup and (**b**) experimental test setup.

The experimental test was performed in a steel loading frame with a capacity of 300 kN. The frame consists of a pair of channel sections placed face-to-face as a horizontal member and two pairs of channel sections placed back-to-back as vertical members. The lateral stability of the loading frame is provided by two additional channel sections on either side of the vertical members for support. The column portion of the specimen was placed between the horizontal member of the loading frame and a steel I-beam that was firmly fixed to the test floor by proper bolt connections. The top of the column was a hinge supported by a steel ball that was inserted into the semi-circular grooves of two steel plates, and the lower end of the column was simply supported. A hydraulic jack was placed in the centre of the steel plates. Using the hydraulic jack, the column was loaded with an initial compressive axial force of 20% of the load-carrying capacity in order to keep the joint stable [62]. Figure 4a illustrates the test setup’s schematic design.

To apply a reverse cyclic load to the joint, a 50 kN hydraulic jack connected to a load cell was used, as shown in Figure 4. Before applying the cyclic loads, the hydraulic jack’s piston was maintained in the centre to achieve both positive and negative loading cycles. As stated earlier, the hydraulic jack was fastened to the I-beam’s top plate, which had also been welded in place. A system of two channel sections and rods were used to transfer the load from the load cell to the beam tip. The entire setup over the load cell was made to move along with the piston when the load was applied. The applied load was noted from the load indicator connected to the load cell. A dial gauge with a 0.01 mm count and a 25 mm travel was used to measure the beam’s deflection at its cantilever end. In order to measure the deformations in the joint, four LVDTs, each with a gauge distance of 200 mm and a least count of 0.001 mm, were utilised. The crack’s width was measured with the help of a crack detection microscope with 40× magnification. The beam-column joint testing is visualised in Figure 4b.

### 2.6. Testing Procedure

The specimens were whitewashed, making it possible to observe the crack patterns more clearly, and loaded in the positive direction up to a particular level. They were then unloaded to the same level of magnitude in the opposite direction and finally reloaded to the initial position to achieve one complete cycle of reverse cycling loading [63]. The magnitude of the loading was increased after each cycle, and this procedure was repeated until the failure of the joints. At the end of every level of applied load, the dial gauge placed at the bottom tip of the beam was used to measure the deflection.

## 3. Results and Discussion

### 3.1. Load-Deflection Behaviour

**Figure 5:**
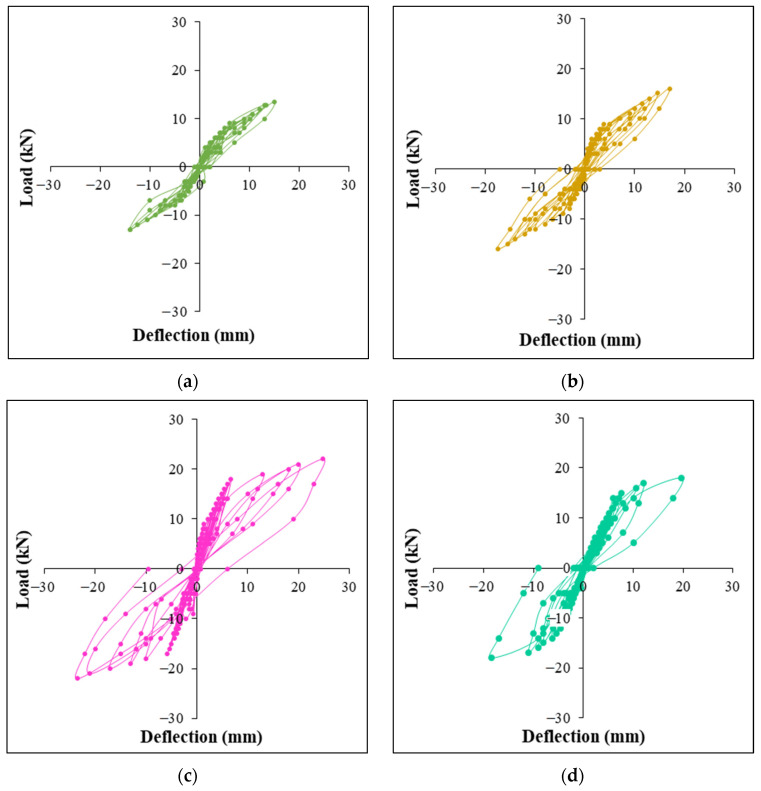
Typical load-deflection plots: (**a**) TGPC, (**b**) HTGPC1, (**c**) HTGPC6 and (**d**) HTGPC8.

**Figure 6:**
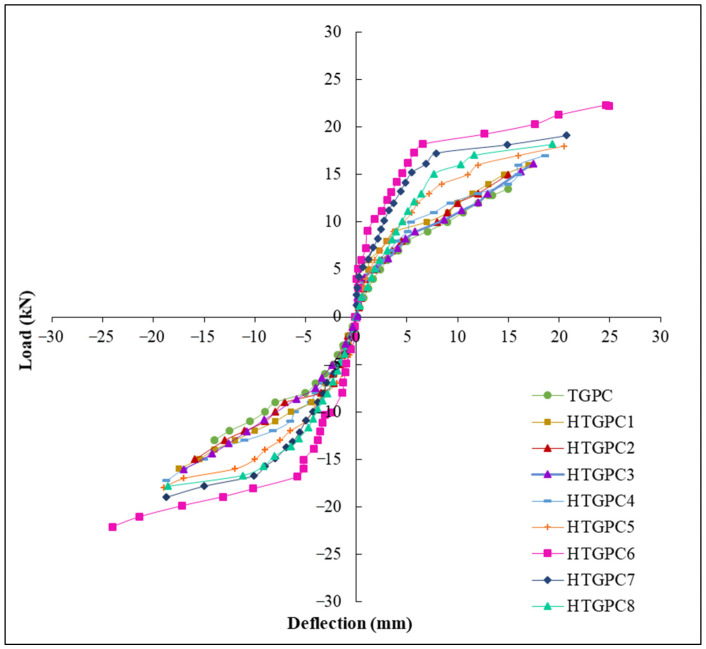
Envelope of load-deflection plots.

The load-deflection curves for the specimens investigated are shown in Figure 5. All of the examined joints’ hystereses are displayed in a single graph (Figure 6) for easier comparison and observation. In order to create the envelope curves, the apex values of all of the load cycles were connected together. It is ascertained from the Figure 6 that when the HTGPC’s hybrid fibre content increases, their ultimate load and the resulting joint deflection improve. This might be associated with the hybrid fibres’ ability to arrest and delay the formation of macrocracks [35]. The growth of microcracks is halted and prevented by the presence of polypropylene fibres near the problem areas. Steel fibres bridge cracks that emerge as a result of the growing magnitude of the load and prevent fissures from extending once they have been formed. However, the strength of joints decreased with increasing volume fractions of polypropylene fibre. This phenomenon may be related to the inability of HTGPC to operate at greater volumes of hybrid fibres, resulting in the balling effect of fibres. A polypropylene fibre concentration of more than 0.15 percent led to the same pattern of findings in several other investigations [24,64].

### 3.2. Moment–Curvature Relationship

**Figure 7:**
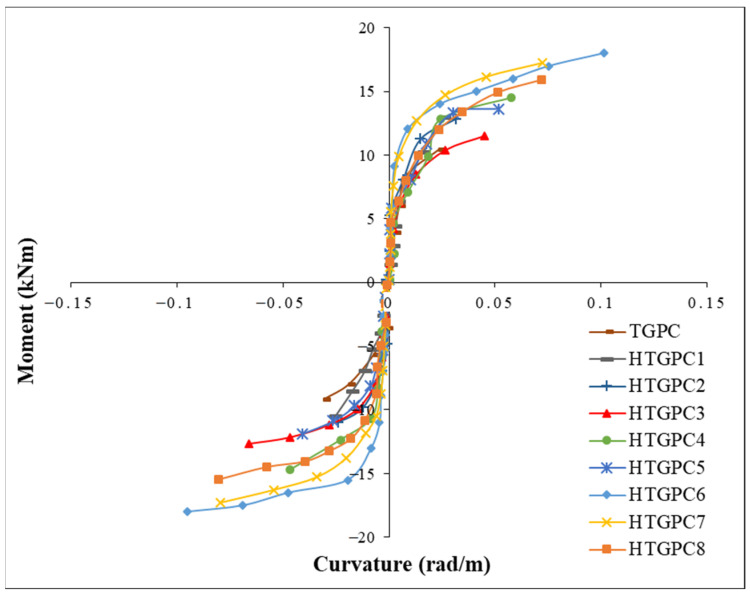
Moment–curvature envelope curves.

The procedure adopted for calculating the moment-curvature relationship was previously explained by the authors elsewhere [65]. The moment-curvature behaviour of a material is crucial for evaluating its ductility property. The measured values for deformations using the top and bottom LVDTs were used to calculate the strain and the corresponding curvatures for plotting the moment-curvature curves. The value of curvature at every stage of loading was calculated for the compression and tension faces to obtain the moment-curvature plots. The envelope curves were obtained by joining the apex values of all the load cycles. Figure 7 shows the envelope curves of all the beam-column joints. It may be observed that the curvature increased with the integration of steel and polypropylene fibres. It has been observed that the curvature regularly increases as the moment increases until the formation of several micro cracks. The plots exhibit a slightly flat trend as the cracks developed further while the specimen reached the peak moment [65]. In the HTGPC specimens, as the steel fibre content increased from 0.5% to 1%, the curvature increased significantly. This demonstrates the contribution of steel fibres to their enhanced ductility. Hence, the HTGPC specimens could perform with a minimal drop in stiffness and strength after their primary yield deformations.

### 3.3. Behaviour of Specimens

**Figure 8:**
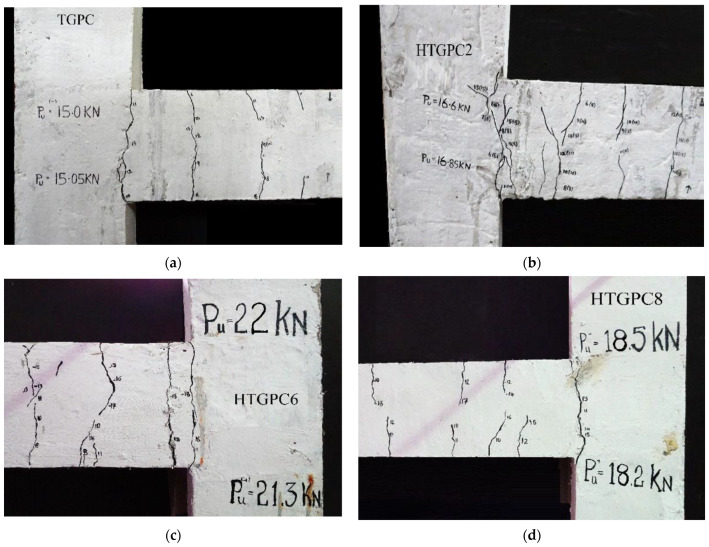
Typical crack patterns of the specimens: (**a**) TGPC, (**b**) HTGPC2, (**c**) HTGPC6 and (**d**) HTGPC8.

Figure 8 depicts the typical failure crack patterns of the TGPC and HTGPC beam-column junctions. All of the specimens developed an initial crack at the point where the beam and the column met, regardless of the fibre content. When the load increased, the micro-cracks widened, and some additional cracks developed on the beam portion. The beam section near the junction had a considerable number of cracks. Finally, the joint failed as the cracks further widened. The cracks were observed to be wider in the TGPC specimens when compared with other HTGPC beam-column joints. During the test, no cracks were generated on the columns, and no joint failure was observed in the tested specimens [66]. In the HTGPC specimens, a greater number of cracks developed, and these were found to be finer than the cracks which developed in the TGPC. This could be associated with the fact that on both micro and macro levels, the combination of steel and polypropylene fibres may be responsible for fracture control. A beam-column joint’s energy absorption can be increased by using steel and polypropylene fibres, which prevents the widening of macrocracks [27]. The first fracture load was determined from the load-deflection envelope plot by comparing it to the point where the initial linearity of the curve diverged [1]. The results of the tests on the beam-column joints may be seen in the table below. Table 3 shows that when the fibre content rose, the initial crack load increased as well. The enhanced tensile strain capacity of the composite near fibres may have been the cause of this [63]. It may be noted that the addition of hybrid fibres increased the initial fracture load by 68 percent and ultimate load by 47 percent for the HTGPC specimen with 1 percent steel and 0.15 percent polypropylene fibres.

### 3.4. Energy Absorption Capacity and Ductility Characteristics

A structure’s load-deflection envelope curve illustrates how much energy it can withstand. Table 3 displays the energy absorption capacity of each specimen investigated. From the Table 3, it may be observed that the energy absorption capacity significantly improved, and an energy absorption capacity nearly three times greater can be observed for the HTGPC specimen which has 1 percent steel fibres and 0.15 percent polypropylene fibres when compared to the TGPC specimen. This demonstrates that specimens with hybrid fibres absorb more energy than specimens without fibres [67].

The capacity of a material to deform past its initial yield deformation while still carrying a load is known as its ductility [63]. If the structure needs to bear repeated lateral strains due to unforeseen conditions, it must have the ability to deform with ductility. The ductility of a structure is measured by comparing its ultimate deflection (δu) to its yield deflection (δy) [68]. The ductility factors for each specimen are presented in Table 3. The table shows that the incorporation of hybrid fibres in concrete influences the ductility factor [1]. According to the table, 2.73 times more energy was absorbed by the HTGPC specimen with 1 percent steel fibre and 0.15% polypropylene fibre when compared to the TGPC specimen without fibres. It was also demonstrated that the HTGPC specimens had a greater deflection at ultimate stress.

### 3.5. Energy Dissipation Capacity

**Figure 9:**
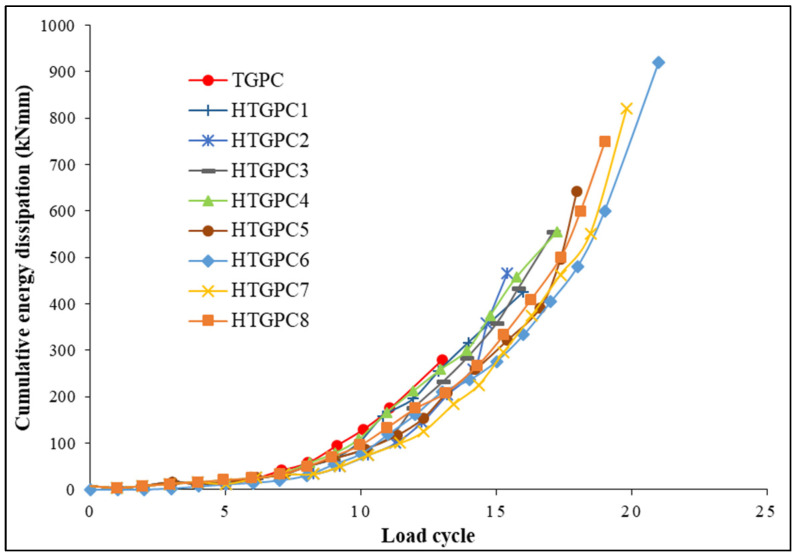
Cumulative energy dissipation of joints.

The hysteresis loop obtained from the load-displacement characteristics was used to obtain the energy dissipation capacity. The area enclosed by this hysteresis loop is expressed as the energy dissipation capacity. The difference between the energy received and released during loading and unloading determines the energy dissipation capability in each loading cycle [63]. A structure’s seismic performance must be evaluated, and structures can dissipate seismic energy only if they can sustain severe ground earthquake motion. Deformations in crucial parts of the structural system provide this energy dissipation, which requires an adequate level of ductility in the components and their joints [69]. In order to calculate the amount of energy lost by the joints during the experiment, the energy dissipated at each load-displacement loop was summed up until the end of the test. The joints’ cumulative energy dissipation measurements during each cycle are provided in Figure 9. The HTGPC specimen absorbed more energy than the TGPC specimen over time, as can be seen in the figure. This demonstrates that the HTGPC specimens with hybrid fibres exhibit enhanced ductile behaviour when compared to TGPC without fibres and can provide sufficient warning before the failure of the structure [63]. The HTGPC specimen with 1 percent steel and 0.15 percent polypropylene fibres dissipated cumulative energy more efficiently than the other specimens.

### 3.6. Stiffness Degradation

**Figure 10:**
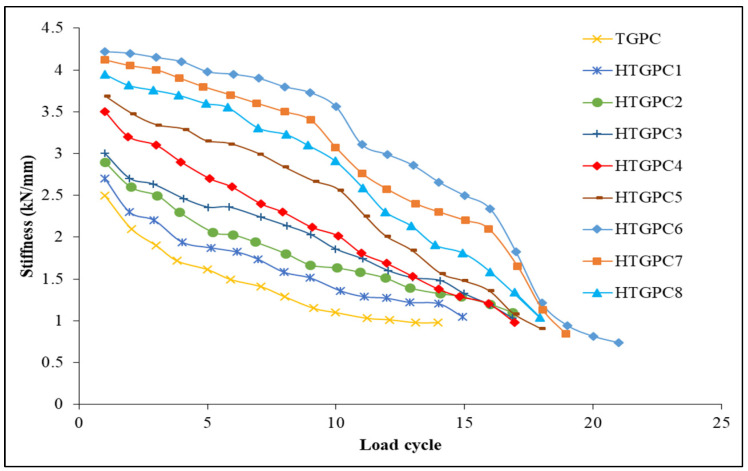
Stiffness degradation plots.

Repetitive or cyclic stress on an RCC beam-column junction reduces the rigidity of the joint. The secant stiffness is used to calculate the stiffness deterioration at the joints, and this may be used to compute the stiffness degradation [70]. The secant stiffness in each cycle was evaluated by drawing a line between the ultimate positive displacement point in one half of the cycle and the ultimate negative displacement point in the other half of the cycle [71]. Stiffness deterioration charts for each specimen are provided in Figure 10. The TGPC joint exhibits the lowest starting stiffness and a dramatic decrease in secant stiffness values, as can be seen in the figure. Since polypropylene has a low modulus of elasticity, it has a very minor effect on the stiffness. Hence, the inclusion of steel fibres may result in a reduction in stiffness degradation when compared to a TGPC specimen [1]. The initial secant stiffness value of the HTGPC joints was effectively enhanced by the integration of hybrid fibres and showed a steady decline in stiffness until the failure of the specimen. The management of fractures at micro and macro levels may be made possible via the effects of hybrid fibres. There were numerous micro-level cracks bridged by polypropylene fibres during the first few loading cycles. For every further loading cycle, these microcracks became wider and were controlled by steel fibres. This effect is designed to limit the growth of cracks and increase the amount of energy needed for fibre pull-out and debonding in the vicinity of cracks. Hybrid fibres in beam-column junctions do not appreciably reduce stiffness during crack development [63].

## 4. Analytical Model for Predicting the Shear Strength of HTGPC Beam-Column Joints

The HTGPC beam-column joints’ shear strength was predicted using the available models in the literature. The specifications of the various models are shown below:

### 4.1. ACI-ASCE Committee 352

The following equation for the nominal joint shear strength of beam-column joints was certified by ACI-ASCE committee 352 [61].

(1) $$V_{n}=\gamma \sqrt{{f^{\prime }}_{c}}b_{j}h_{c}$$

where:

$V_{n}$ = the nominal shear strength of the joint, in N;
${f^{\prime }}_{c}$ = the concrete cylinder strength, in MPa;
$b_{j}$ = the effective joint width, in mm;
$h_{c}$ = the depth of the column, in mm;
γ = for the interior, exterior and corner joints, the confinement by the members’ frame are rated at 1.67, 1.25 and 1.0, respectively.

### 4.2. AIJ Guidelines

The following equation was developed by the Architectural Institute of Japan [72] to determine joint shear strength.

(2) $$V_{j}=k\vartheta F_{j}b_{j}h_{c}$$

where:

$V_{j}$ = the ultimate shear strength of the joint;
k = the shape of the joint factor (1.0 for cross shape and 0.7 for T-shape joints);
ϑ = 0.85 for T-shape joints;
$F_{j}$ = $0.8\times f_{ck}{}^{0.7}$, in MPa;
$f_{ck}$ = the concrete’s compressive strength, in MPa

### 4.3. Bakir

Based on the studies carried out by Bakir [73], an equation for joint shear strength was obtained and is given by,

(3) $$V_{j}=\left(\frac{b_{c}+b_{b}}{2}\right)h_{c}\lambda \left(0.092{f^{\prime }}_{c}+0.55\ln\left(\frac{h_{c}}{d_{b}}\right)+0.23\;\frac{A_{sh}f_{ys}}{\left(\frac{b_{b}+b_{c}}{2}\right)h_{c}}\right)$$

where:

$b_{c}$ = the width of the column, in mm;
$b_{b}$ = the width of the beam, in mm;
$d_{b}$ = the diameter of the beam longitudinal reinforcement, in mm;
*λ* = a capacity reduction factor of 0.78;
$f_{ys}$ = the yield strength of transverse reinforcement, in MPa;
$A_{sh}$ = the area of shear reinforcement in the joint, in mm^2^.

### 4.4. Jiuru et al.

Based on the concept that even after cracking, the composite still retains considerable tension until fibres are pulled from the matrix, a model was constructed to predict ultimate shear strength [62]. The equation provided is,

(4) $$V_{j}=V_{c}+V_{f}+V_{s}$$

where:

$V_{c}$ = the shear carried by the concrete = $0.1\;\left(1+\frac{N}{b_{c}h_{c}f_{ac}}\right)b_{j}h_{j}f_{ac}$;
*N* = the axial compressive load of the column, in N;
$f_{ac}$ = the axial compressive strength of the concrete, in MPa;
$h_{j}$ = the effective joint depth, in mm
$V_{f}$ = the shear carried by the fibres = $2\frac{l_{f}}{d_{f}}V_{f}b_{j}h_{j}$;
  $l_{f}$ = the length of steel fibres, in mm;
$d_{f}$ = the diameter of steel fibres, in mm;
$V_{f}$ = the volume percentage of steel fibres;
$V_{s}$ = the shear carried by the joint stirrups = $f_{ys}\frac{A_{sh}}{S}(d-d^{\prime }$);
*D* = the effective depth of the beam, in mm;
d′ = the effective cover to compressive reinforcement, in mm.

### 4.5. Tsonos

After a series of investigations, Tsonos [74] came up with a design based on the strut and tie concept. The equation may be used to calculate joint shear strength is,

(5) $${\left(\frac{\alpha \lambda }{2\sqrt{f_{ci}}}\left(1+\sqrt{1+\frac{4}{\alpha^{2}}}\right)\right)}^{5}+\frac{5\alpha \lambda }{\sqrt{f_{ci}}}\left(\sqrt{1+\frac{4}{\alpha^{2}}}-1\right)=1$$

where:

$f_{ci}=1+\frac{\rho_{s}f_{s}}{{f^{\prime }}_{c}}$;
  $\rho_{s}=$ the volume ratio of the transverse hoop reinforcement;
$f_{s}=$ the yield strength of the transverse hoop reinforcement, in MPa;
$\alpha =\frac{h_{b}}{h_{c}}$;
$h_{b}=$ the depth of the beam, in mm;
λ= the joint shear stress, expressed as a multiple of $\sqrt{f_{c}}$;

This Equation was developed based on the basis of the hypothesis that the strength of the core concrete is directly proportional to the strength of the strut and truss mechanisms, respectively.

Comparison of analytical models with the experimental results:

The shear strength of the joints was calculated for the TGPC and HTGPC specimens using the above equations, and a comparison of the experimental results is provided in Table 4. According to the authors’ extensive experimental work, the mechanical characteristics of the TGPC with hybrid fibres were determined. The results of this study are reported in a separate article [24].

Table 4 shows that the average and coefficient of variation imply that the comparison is inadequate. There is a chance that this is because the equations in the literature examined for the comparison are for either normal concrete or fibre-reinforced normal concrete, which makes it impossible to draw a true parallel. In the literature, hybrid fibres and TGPC are not taken into account for the models. Since HTGPC contains steel and polypropylene fibres, the equation requires a correction factor. Table 4 shows that the model suggested by Jiuru et al. [62] has the lowest coefficient of variation when compared to the other equations. As a result, an attempt was made to alter the design model.

### 4.6. Modification Proposed

Regression analysis was used in the model developed by Jiuru et al. [62] to account for the influence of steel and polypropylene fibres. In the process of creating a correction factor, numerous factors such the compressive strength, flexural strength and contribution of steel and polypropylene fibres were considered and is given by,

(6) $$F_{j}=\left(A_{fs}V_{s}\eta_{bs}+A_{fp}V_{p}\eta_{bp}\right)\frac{f_{cr}}{f_{c}}$$

**Figure 11:**
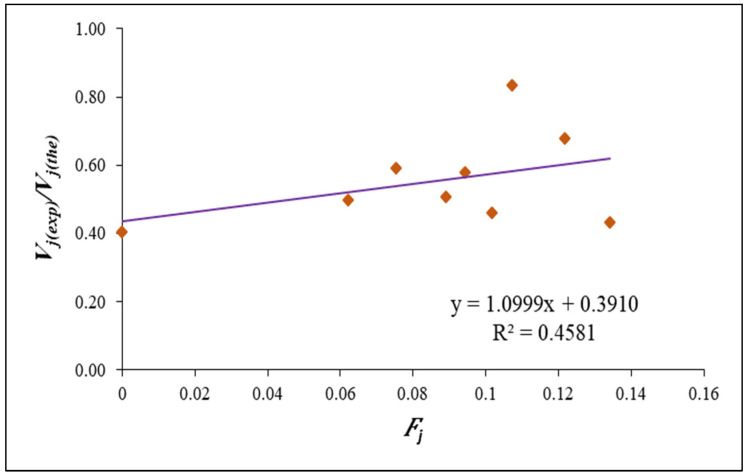
Correction factor (*F_j_*) against $V_{j\left(\exp\right)}/V_{j\left(\mathrm{the}\right)}$.

The values of $F_{j}$ were plotted against $V_{j\left(\exp\right)}/V_{j\left(\mathrm{the}\right)}$, as shown in Figure 11. The regression equation thus obtained for the best fit line of the plot is,

(7) $$V_{j\left(\exp\right)}/V_{j\left(\mathrm{the}\right)}=\left(1.0999F_{j}+0.3910\right)$$

As the experimental shear strength should be equal to the corrected shear strength, $V_{j\left(\exp\right)}$ is replaced by $V_{j\left(\mathrm{pre}\right)}$, and thus Equation (7) becomes

(8) $$V_{j\left(\mathrm{pre}\right)}=V_{j\left(\mathrm{the}\right)}\;\left(1.0999F_{j}+0.3910\right)$$

**Figure 12:**
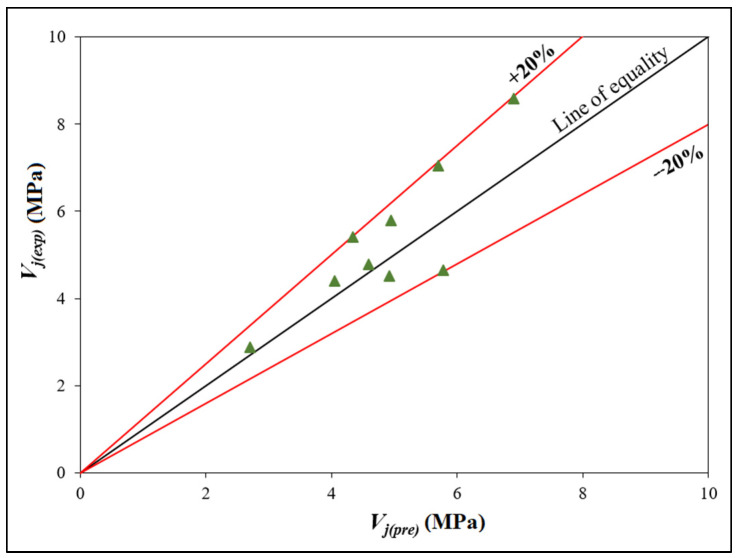
Comparison of experimental and predicted values for the shear strength of joints.

It is shown in Figure 12, the joint’s shear strength is compared to its predicted strength. The graph demonstrates that all points are within a ±20% agreement range of the line of equality. As a result, the suggested model accurately predicts the shear strength of HTGPC joints.

## 5. Conclusions

This experimental investigation leads to the following conclusions:

1. The first crack load, ultimate strength, ductility, stiffness degradation and energy dissipation capacity were improved with the incorporation of hybrid fibres in TGPC. This shows that the fibres in a hybrid form can be effectively used in TGPC.
2. The first crack loads of HTGPC joints with a constant volume of steel fibres of 1% and a varied volume fraction of fibres of 0.10%, 0.15%, 0.20% and 0.25% are 33%, 68%, 53% and 36%, respectively, making them higher than that of TGPC joints without fibres.
3. The energy absorption capacity and the ductility of the HTGPC6 were improved by 3.3 times and 2.73 times, respectively, compared to TGPC without fibres.
4. The cumulative energy dissipation and the stiffness degradation of the HTGPC6 were improved by a maximum of 3.7 and 1.6 times, respectively, when compared to the TGPC specimen.
5. HTGPC with 1.0% steel and 0.15% polypropylene fibres demonstrated the maximum deflection at the ultimate load and performed better than the other combinations considered in this study. The deterioration in the performance with the further addition of fibres resulted from poor workability and a balling effect at higher volume fractions.
6. Hybrid fibres can reduce steel reinforcement congestion in HTGPC beam-column junctions and ease construction difficulties, resulting in cost-effective construction.
7. HTGPC beam-column joints with a maximum content of 1% steel and 0.25% polypropylene fibres may be predicted using the proposed equation. HTGPC structures will benefit from the findings of this investigation’s tests.
8. HTGPC can be used as the superior alternative for conventional cement concrete structures which are required to withstand unforeseen situations such as seismic and wind loads.

## Figures and Tables

**Table 1:** Mix proportions of HTGPC mixes.

Mix ID	Fly Ash	GGBS	MK	Fine Aggregate	Coarse Aggregate	Na_2_SiO_3_	NaOH	Water	SP	SF	PF
	Kg/m^3^										
TGPC	237.47	122.61	64.53	554.40	1293.60	90.99	36.40	84.92	6.37	-	-
HTGPC1	237.47	122.61	64.53	554.40	1293.60	90.99	36.40	84.92	6.37	39.25	0.95
HTGPC2	237.47	122.61	64.53	554.40	1293.60	90.99	36.40	84.92	6.37	39.25	1.425
HTGPC3	237.47	122.61	64.53	554.40	1293.60	90.99	36.40	84.92	6.37	39.25	1.90
HTGPC4	237.47	122.61	64.53	554.40	1293.60	90.99	36.40	84.92	6.37	39.25	2.375
HTGPC5	237.47	122.61	64.53	554.40	1293.60	90.99	36.40	84.92	6.37	78.50	0.95
HTGPC6	237.47	122.61	64.53	554.40	1293.60	90.99	36.40	84.92	6.37	78.50	1.425
HTGPC7	237.47	122.61	64.53	554.40	1293.60	90.99	36.40	84.92	6.37	78.50	1.90
HTGPC8	237.47	122.61	64.53	554.40	1293.60	90.99	36.40	84.92	6.37	78.50	2.375

**Table 2:** Properties of reinforcing bars.

Nominal dia. of Bar, mm	Actual dia. of Bar, mm	Yield Strength, MPa	Ultimate Strength, MPa	Young’s Modulus, GPa
10	9.95	530	582	230
6	6.12	528	579	225

**Table 3:** Test results for beam-column joints.

Mix ID	First Crack Load (kN)	Ultimate Load (kN)		Deflection at Ultimate Load (mm)		Energy Absorption (kNm)		Ductility Factor
		Forward Cycle	Reverse Cycle	Forward Cycle	Reverse Cycle	Forward Cycle	Reverse Cycle	
TGPC	6	15.05	15.00	13.8	14.3	0.135	0.141	1.41
HTGPC1	6.5	16.80	16.30	18.6	18.8	0.213	0.206	1.63
HTGPC2	7	16.85	16.60	16.0	16.5	0.251	0.243	2.15
HTGPC3	7.8	16.80	17.00	16.2	16.5	0.269	0.271	2.19
HTGPC4	8.5	17.20	17.25	16.9	17.5	0.309	0.288	2.21
HTGPC5	8	18.00	18.30	19.6	19.1	0.315	0.308	2.62
HTGPC6	10.1	21.30	22.00	24.8	23.5	0.457	0.417	3.85
HTGPC7	9.2	19.05	20.02	20.8	18.5	0.414	0.389	3.42
HTGPC8	8.2	18.20	18.50	20.5	19.0	0.368	0.335	3.05

**Table 4:** Experimental and theoretical values comparison.

Specimen	*V_j_*_(exp)_, MPa	*V_j_*_(the)_, MPa					*V*_*j*(exp)_/*V*_*j*(the)_				
		ACI-ASCE	AIJ	Bakir	Jiuru et al. [62]	Tsonos	Ratio	Ratio	Ratio	Ratio	Ratio
	i	ii	iii	iv	v	vi	i/ii	i/iii	i/iv	i/v	i/vi
TGPC	2.88	8.47	8.10	4.75	7.13	9.43	0.34	0.36	0.61	0.40	0.31
HTGPC1	4.39	8.77	8.50	4.99	8.84	10.09	0.50	0.52	0.88	0.50	0.44
HTGPC2	5.41	8.79	8.53	5.00	9.17	10.14	0.62	0.63	1.08	0.59	0.53
HTGPC3	4.77	8.75	8.48	4.97	9.41	10.05	0.55	0.56	0.96	0.51	0.48
HTGPC4	4.52	8.82	8.58	5.03	9.82	10.22	0.51	0.53	0.90	0.46	0.44
HTGPC5	5.78	9.15	8.98	5.30	10.03	10.84	0.63	0.65	1.09	0.58	0.53
HTGPC6	8.57	9.07	8.92	5.23	10.26	10.84	0.95	0.96	1.64	0.84	0.79
HTGPC7	7.05	8.95	8.76	5.14	10.38	10.52	0.79	0.81	1.37	0.68	0.67
HTGPC8	4.65	9.00	8.82	5.18	10.76	10.63	0.52	0.53	0.90	0.43	0.44
Average							0.60	0.62	1.05	0.55	0.51
Coefficient of variation (%)							29.5	28.9	28.8	24.5	27.8

## Data Availability

Not applicable.

## Nomenclature

$\eta_{bs}$	bond factor for steel fibre
$\eta_{bp}$	bond factor for polypropylene fibre
*f_cr_*	flexural strength of concrete
*f_c_*	compressive strength of concrete
*A_fs_*	aspect ratio of steel fibre
*A_fp_*	aspect ratio of polypropylene fibre
*F_j_*	fibre correction factor
*V_s_*	volume fraction of steel fibres
*V_p_*	volume fraction of polypropylene fibres
*V_j_*	ultimate shear strength of joint
